# Role of rGO in improving the mechanical and thermal performance of PANI/graphene nanocomposites

**DOI:** 10.1038/s41598-025-29897-0

**Published:** 2025-12-07

**Authors:** Zeeshan Mustafa, Dhruva Kumar, Bibhu Prasad Swain, B. B. Pradhan, Ranjan Kumar Ghadai

**Affiliations:** 1 Department of Mechanical Engineering, Sikkim Manipal Institute of Technology, Sikkim Manipal University, Majitar, 737136 Sikkim India; 2 Department of Physics, National Institute of Technology Manipur, Langol, Imphal, 795004 Manipur India; 3 Department of Mechanical Engineering, Sikkim Institute of Science and Technology, Chisopani, 737121 Sikkim India; 4 Department of Mechanical and Industrial Engineering, Manipal Institute of Technology, Manipal Academy of Higher Education, Manipal, 576104 Karnataka India

**Keywords:** Polyaniline (PANI), Reduced graphene oxide (rGO), Tribology, Nanoscratch, Thermo-mechanical stability, Thermogravimetric analysis, Engineering, Materials science, Nanoscience and technology

## Abstract

Reduced graphene oxide (rGO) is a potential reinforcement for polyaniline (PANI) owing to its high stiffness, thermal stability, and intrinsic lubricity. Considering this, the present study investigates the effect of rGO content on the mechanical, tribological, and thermal properties of Polyaniline/rGO (PANI/G) nanocomposites (NCs) synthesized via in-situ oxidative polymerization with systematically varied rGO content (0.01–0.10 wt%). The samples were then characterized using Scanning Electron Microscopy and Energy Dispersive X-ray Spectroscopy (EDX). Nanoindentation and nanoscratch testing revealed that hardness, modulus, and wear resistance enhanced with increasing rGO content, while the elastic-plastic indices (H/E and H^3^/E^2^) decreased, suggesting a trade-off between stiffness and recoverability. Complementary thermogravimetric analysis (TGA) demonstrated that higher rGO loading (0.10 wt%) improved thermal stability by delaying the onset of degradation and reducing overall weight loss, underscoring the multifunctional reinforcing attribute of rGO. The combined results established that an optimized rGO concentration not only enhances surface mechanical integrity but also significantly improves resistance against thermal decomposition, making PANI/G NCs a potential candidate for structural, protective coating, and high-temperature electronic applications.

## Introduction

Polymer nanocomposites (PNCs) have emerged as a versatile class of engineered materials capable of bridging the performance gap between structural composites and conventional polymers^1^. Through the incorporation of nanoscale reinforcements, polymers can acquire enhanced stiffness, strength, toughness, and multifunctionality while retaining low density and facile processability. Such attributes make PNCs potential candidates for coatings, electronic devices, packaging, and structural components^2–4^. Amongst the variety of conducting polymers, polyaniline (PANI) has gained prominent attention due to its reversible doping/de-doping behavior, tunable electrical conductivity, ease of synthesis, stability, and environmentally benign nature^5^. These factors make PANI an appealing candidate for applications ranging from energy storage and sensing to anticorrosive coatings and flexible electronics^3,6,7^.

Despite these advantages, pristine PANI often suffers from inherent mechanical limitations, such as moderate stiffness, poor wear resistance, and susceptibility to crack formation under cyclic loading. Consequently, using PANI in mechanically demanding applications becomes limited. To overcome this drawback, reinforcing PANI with nanostructured fillers has been a widely adopted strategy^8^. Among potential nanofillers, graphene and its derivatives, graphene oxide (GO) and reduced graphene oxide (rGO), especially reduced graphene oxide (rGO), are particularly promising owing to their exceptional intrinsic properties. GO is a highly oxidised form of graphene bearing abundant oxygenated functional groups (hydroxyl, epoxy, carboxyl, etc.), which enhances its dispersibility in polar media and facilitates interfacial interactions with polymers. However, these oxygen functionalities interrupt the sp² conjugated carbon network, reducing electrical conductivity, intrinsic stiffness, and thermal stability. In contrast, rGO arises from partial reduction of GO. Many of the oxygen groups are removed, restoring sp² domains, and thereby improving conductivity, mechanical rigidity, and thermal behaviour, while still retaining enough functional groups to aid interfacial adhesion^9,10^.

Also, rGO possesses a Young’s modulus of approximately 1 TPa and an intrinsic strength exceeding 100 GPa, in addition to an extremely high aspect ratio and specific surface area^11,12^. Furthermore, graphene sheets display lamellar sliding under shear, giving them an inherent lubricating ability^13^. These attributes position rGO as a dual-function additive comprising of a load-bearing reinforcement and a solid lubricant within polymer matrices.

Literature reports have primarily concentrated on the electrochemical benefits of PANI/G NCs, particularly in the field of supercapacitors, corrosion protection, and energy storage. For example, Zhang et al.^6^ and Joo *et* al.^7^ individually demonstrated substantial improvements in capacitance and cyclic stability of PANI/G composites. Likewise, Yang et al.^14^ and Fazli-Shokouhi et al.^15^ reported on the anticorrosive behavior of PANI/graphene oxide coatings, highlighting graphene’s barrier properties. While such studies provide valuable insights into the electrochemical and protective functionalities of PANI/G composites, comparatively fewer systematic studies have addressed their mechanical robustness, wear resistance, and thermo-mechanical stability. However, recent reports have begun to underscore graphene’s role in enhancing the mechanical and tribological performance of polymer composites. For instance, Maurya et al.^16^ investigated PANI/G coatings on magnesium alloys and observed improved hardness and corrosion resistance. Dayyoub et al.^17^ reported improved wear resistance in PANI containing UHMWPE/graphene systems. These findings collectively suggest that graphene incorporation not only enhances strength but also modifies surface interaction during sliding, thereby reducing material loss. Nevertheless, a comprehensive understanding of how a systematic variation of rGO content influences the mechanical, tribological, and thermal performance of PANI/G NCs is required.

A suitable approach to characterizing such composites is instrumented nanoindentation, as it provides direct measurements of hardness (H) and elastic modulus (E). In addition to these primary parameters, elastic-plastic indices, such as H/E and H^3^/E^2^ have been widely employed as compact descriptors of material performance in contact mechanics^18^. Herein, the ratio H/E is considered a measure of elastic strain to failure, reflecting the material’s ability to elastically accommodate load before yielding^19^. In the meantime, the ratio H^3^/E^2^ is correlated with resistance to plastic deformation, serving as an indicator of durability against permanent indentation or wear^20^. Although these indices have been extensively explored for coatings and thin films, their role in understanding the thermo-mechanical behavior of PNCs, particularly PANI/G, has not been adequately studied. Additionally, thermal stability is a crucial parameter for multifunctional applications such as electronics and coatings. The incorporation of graphene or rGO is known to enhance polymer thermal resistance by functioning as a barrier to volatile degradation products and facilitating heat dissipation^21,22^.

A few recent studies have attempted multi-property optimization in PANI/rGO systems. For instance, Boublia et al. (2024) employed response surface methodology and artificial neural networks to optimize multifunctional PANI/rGO nanocomposites, though their focus was predominantly on electrochemical behavior rather than mechanical or thermal stability^23^. Likewise, Xue et al. (2025) demonstrated that even low rGO loadings can significantly modulate thermoelectric and mechanical performance; however, the interplay of nanoindentation-derived elastic–plastic indices (H/E, H³/E²), wear resistance, and degradation kinetics has not been systematically examined^24^. These gaps underscore the need for a holistic approach that not only identifies the optimum filler content but also reveals the trade-offs between stiffness, recoverability, wear durability, and thermal robustness in PANI/rGO nanocomposites.

In this regard, the current study aims to fill these gaps by systematically investigating the effect of rGO content (0.01–0.10 wt%) on the mechanical, tribological, and thermo-mechanical properties of PANI/G NCs. The selected loading range of 0.01–0.10 wt% was based on prior literature, where it was observed that even a very small rGO additions were shown to produce significant changes in polymer composite properties due to the extremely high aspect ratio and surface area of graphene-based fillers. Beyond ~ 0.10 wt%, most studies have reported severe agglomeration of rGO nanosheets, which hinders uniform dispersion and deteriorates composite performance^24,25^.

## Materials and methods

### Material synthesis

**Fig. 1:**
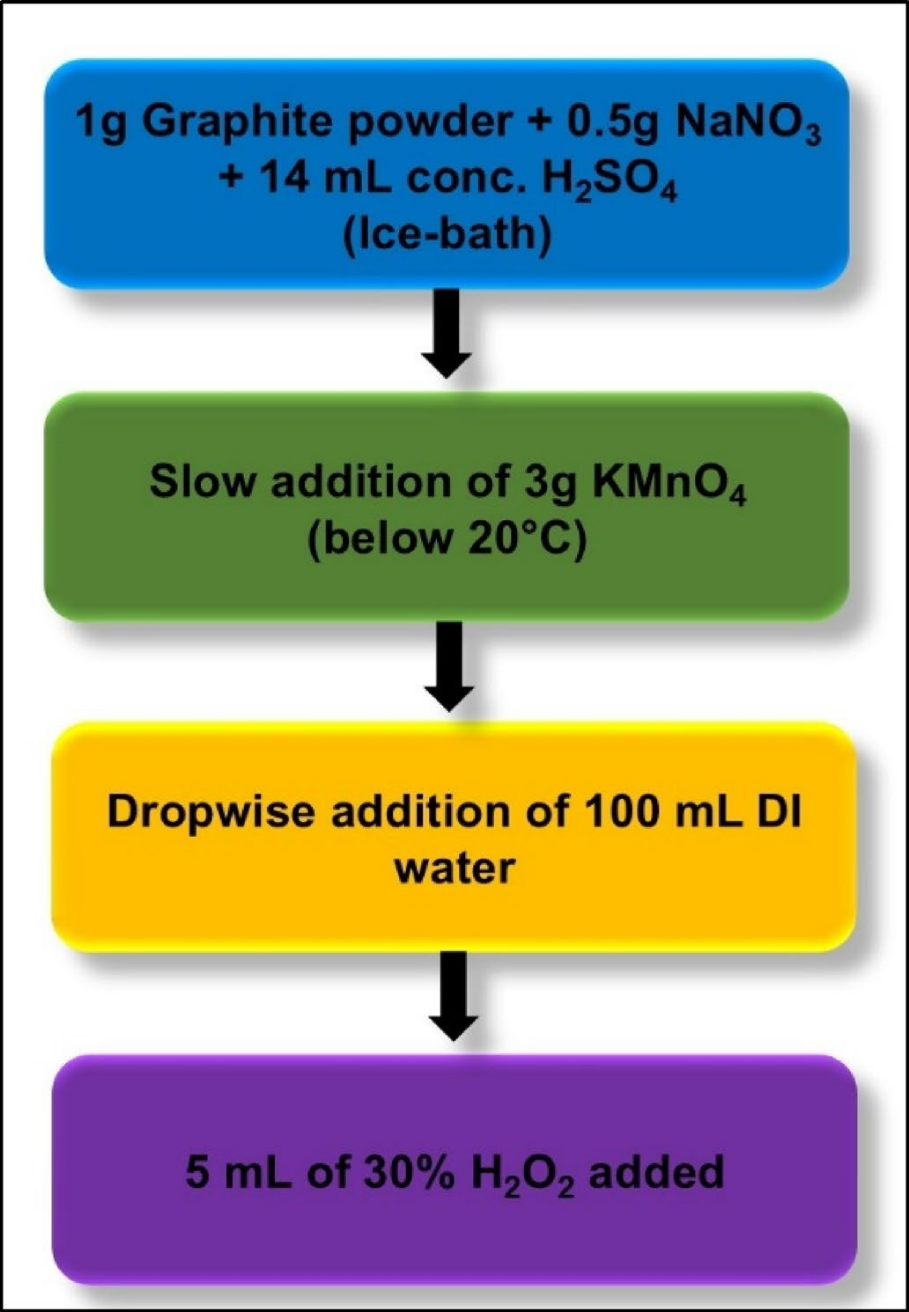
Flowchart summarizing rGO synthesis using modified Hummer’s method.

In this study, rGO was synthesized via the modified Hummers’ method^26^. The synthesis procedure has been depicted in the flowchart given in Fig. 1. Briefly, to prepare rGO, 1 g of graphite powder was mixed with 0.5 g of sodium nitrate (NaNO_3_) and 14 mL of concentrated sulphuric acid (H_2_SO_4_) in an ice bath. To this, 3 g of potassium permanganate (KMnO_4_) was gradually added while maintaining the temperature below 20 °C. The mixture was stirred for 3 h, and then 100 mL of deionized (DI) water was added dropwise. The temperature was then raised to ~ 90 °C. Thereafter, 5 mL of 30% hydrogen peroxide (H_2_O_2_) was added to terminate the reaction. The resulting precipitate was then filtered and washed with 10% HCl and DI water. The rGO so obtained was then dispersed in water and reduced using 2 mL hydrazine hydrate at 90 °C for 1 h.

For the synthesis of PANI/G NCs, 0.01 g rGO (obtained through the aforementioned procedure) and 1 mL of aniline were dispersed in 27.8 mL of 5 M H_2_SO_4_ and ultrasonicated for 1 h. Another solution was prepared by dissolving 2.5 g APS in 27.8 mL of 0.5 M H_2_SO_4_ solution. This solution was added to the rGO-aniline dispersion under ice-bath conditions with constant stirring. The stirring continued for 8 h. The resulting dark precipitate of PANI/G-0.01 NCs was filtered, washed, and dried. Likewise, 0.03, 0.05, 0.07, and 0.10 wt% rGO were used to prepare PANI/G-0.03 NCs, PANI/G-0.05 NCs, PANI/G-0.07 NCs, and PANI/G-0.10 NCs, respectively. Each composition of PANI/rGO nanocomposites was synthesized in triplicate batches under identical conditions to ensure reproducibility. The measured mechanical, tribological, and thermal properties were consistent across batches, with variations within approximately 5% of the mean values.

### Nanoindentation testing

The mechanical properties of the PANI/G nanocomposite were examined using the nano-indentation technique (TI 950, Hysitron Inc., USA) with a standard Berkovich indenter tip and a nano-tribological testing facility and an in-situ SPM (Scanning Probe Microscopy) imaging facility. To maintain a quasi-static loading regime and reduce the impact of strain rate or thermal drift, the loading rate was set at 50–100 µN/s, and the unloading rate was set at 100–200 µN/s. The maximum applied load was 1000 µN. According to the Oliver & Pharr approach, it was ensured that the maximum penetration depth during indentation did not surpass 10% of the thickness^27^. A standard quartz sample, which accounts for tip rounding and guarantees precise contact area estimation, was used to calibrate the tip area function^28^. A conventional post-unloading drift correction approach was used to detect thermal drift. The indenter was kept in contact with the sample for 60 s following each indentation cycle at a modest load (about 10% of the maximum). The displacement data was corrected across the whole load-displacement curve using the rate of depth change that was noted during this time. Following at least 45 min of thermal stabilization, all tests were carried out, and the drift rates recorded fell within the permissible range specified in the literature at less than 0.05 nm/sec. This adjustment guarantees that mechanical property measurements are accurate, especially when the indentation depth is shallow. On various parts of the sample surface, at least ten indentations were made at each stress level. In order to reduce substrate effect and stress field overlap, the distance between two successive indentations was kept at least ten times the maximum indentation depth. By doing this, the precision and dependability of the mechanical property data were maintained since each indentation was guaranteed to be independent and unaffected by nearby observations. Young’s modulus (E) and Hardness (H) mean values were considered in order to examine other mechanical properties. The thickness was measured using Dektak profilometer (model no. Dektak V300), and it was found to vary in the range of 2.3 μm to 6.6 μm for different rGO content (0.01–0.10 wt%).

### Nanoscratch testing

Nanoscratch experiments were carried out to assess the adhesion and wear resistance of the NCs surfaces. A ramped normal load was applied while the indenter was dragged laterally across the film’s surface. The resulting scratch tracks were analyzed to evaluate the coefficient of friction and wear rate.

### Thermogravimetric analysis (TGA) testing

Thermogravimetric analysis (TGA) was conducted to evaluate the thermal behavior of the as-synthesized samples. The measurements were carried out employing a Perkin Elmer STA 6000 instrument under a controlled nitrogen atmosphere in order to restrict oxidation during heating. Approximately 10 mg of each sample was weighed and placed in a platinum crucible. The analysis was performed at a heating rate of 10 °C/min, with a nitrogen gas flow of 20 mL/min maintained throughout the experiment to ensure an inert environment.

### Scanning electron microscopy (SEM) and energy dispersive X-ray (EDX) spectroscopy

The SEM and EDX characterizations of the samples were performed using EVO MA18 with Oxford EDS (X-act).

## Results and discussion

### Morphological and elemental analysis

**Fig. 2:**
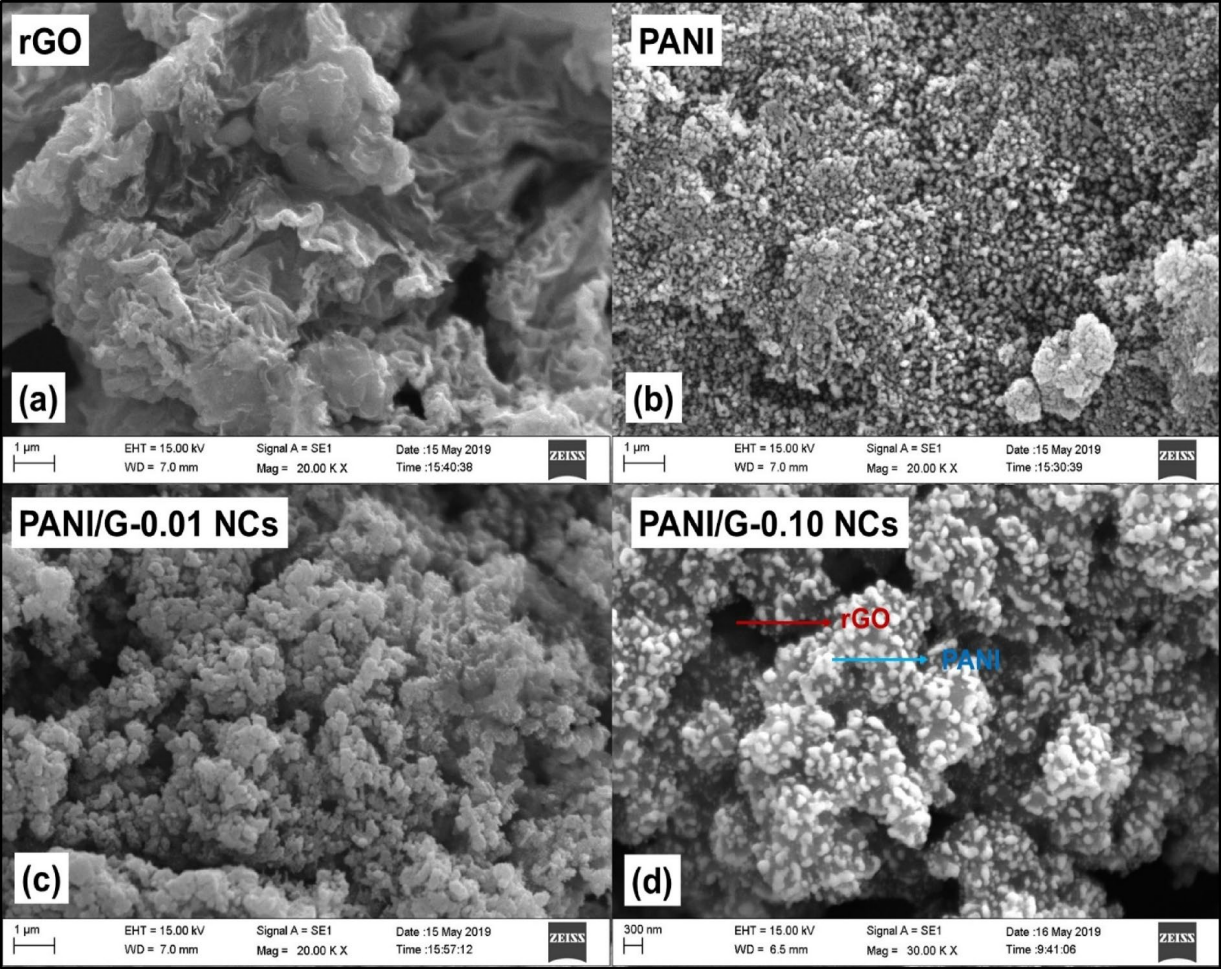
SEM images of (**a**) rGO, (**b**) PANI, (**c**) PANI/G-0.01 NCs, and (**d**) PANI/G-0.10 NCs.

SEM analysis was employed to interpret the surface morphologies of the samples. Figure 2a represents the SEM image of rGO. As may be observed from the figure, the rGO sheets displayed the characteristic wrinkled and crumpled structure, which arises due to partial reduction and restacking of graphene nanosheets. Such sheet-like features are consistent with reported Literature reports, where exfoliated rGO nanosheets show folds and ridges that enhance surface roughness and provide anchoring sites for polymer chains^29,30^. These wrinkled surfaces are especially beneficial in NCs formation, as they improve interfacial contact and prevent polymer chain mobility, thereby contributing to improved mechanical and thermal stability.

Figure 2b shows the morphology of pristine PANI. The observed morphology exhibited granular and agglomerated particles, which are typical of PANI synthesized via the oxidative polymerization method^31^. The observed granular morphology results from the heterogeneous nucleation and growth during polymerization, and has been reported to greatly influence electrochemical activity and charge transport properties^32^. However, pristine PANI inclines towards the formation of irregular aggregates, resulting in poor packing density and restricted structural integrity under stress. Figure 2c illustrates the SEM image of PANI/G-0.01 NCs. At this rGO content, rGO sheets partially coated with PANI particles, but the dispersion of graphene within the matrix appeared limited.

Earlier reports have suggested that insufficient graphene loading results in suboptimal filler-polymer interaction, causing partial agglomeration of rGO sheets and a discontinuous stress-transfer pathway^33^. Nevertheless, the presence of rGO at this concentration already introduces nucleation sites for PANI growth, producing smaller and more uniformly distributed particles compared to pristine PANI. Figure 2d depicts the morphology of PANI/G-0.10 NCs which shows a more uniform and compact morphology. Herein, the PANI matrix appeared to be intimately wrapped around the rGO sheets, leading to strong interfacial adhesion and reduced void content. This suggests that an optimum concentration of rGO enhances the dispersion of filler and promotes synergistic interaction between the polymer chain and nanosheets. Such compact morphologies have been correlated in earlier report with improved hardness, wear resistance, and thermal stability^34^. Thus, these morphological observations confirm that the incorporation of rGO significantly modifies the morphological features of PANI, transitioning it from a loosely aggregated granular form to a well-packed composited network at optimized filler loadings.

**Fig. 3:**
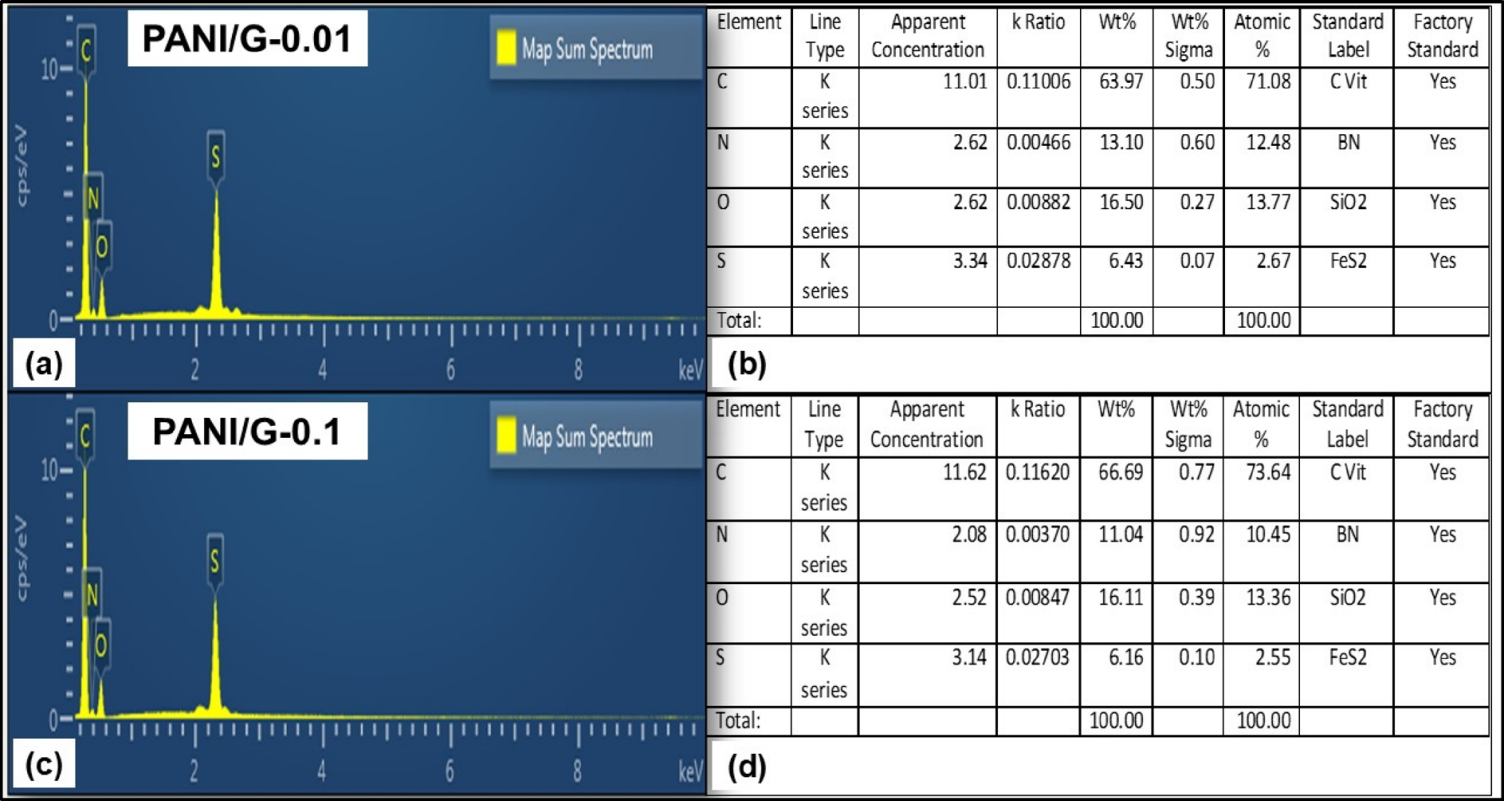
EDX spectra of (**a**) PANI/G-0.01 NCs, (**b**) Percentage composition of the elements in PANI/G-0.01 NCs, (**c**) PANI/G-0.10 NCs, and (**d**) Percentage composition of the elements in PANI/G-0.1 NCs.

EDX analysis was used to verify the elemental composition of the as-synthesized samples. Figure 3a shows the EDX spectra of PANI/G-0.01 NCs, while Fig. 3b represents the percentage composition of the elements. Figure 3c shows the EDX spectra of PANI/G-0.10 NCs with Fig. 3d displaying the percentage composition of the elements. From the spectra, distinct peaks corresponding to carbon (C), nitrogen (N), oxygen (O), and sulfur (S) may be observed. Here, the dominant C peak originates from both the PANI matrix and the rGO nanosheets, whereas the N signal is a characteristic peak of the quinoid/benzenoid units of PANI. The presence of O indicates incomplete reduction of graphene oxide and residual oxygenated functional groups, which are known to improve interfacial compatibility with the polymer chain^29,30^. The S peak may be ascribed to the dopant anions introduced during oxidative polymerization using HCl and APS, reflecting successful incorporation of PANI^31^.

As described previously, a similar elemental profile was detected; however, the relative intensity of the C peak was significantly enhanced compared to the 0.01 wt% composition, which reflects the higher proportion of carbon-rich filler within the analyzed area. It is important to note that these variations in elemental intensities arise from differences in filler content and spatial distribution, rather than any alteration in the intrinsic chemical composition of the nanocomposites. The retention of N and S peaks across all samples indicates effective doping and uniform distribution of PANI. Notably, the O peak intensity decreased relative to C, which indicates more efficient reduction of graphene oxide at higher filler concentrations and reduced oxygen-rich defects^32^. Since EDX is a semi-quantitative technique, the observed elemental variations indicate relative trends due to filler loading and spatial distribution, rather than new chemical bonding. It should be noted that EDX primarily confirms elemental composition and relative filler content; interfacial interactions are inferred indirectly from SEM morphologies, mechanical and tribological responses, and improved thermal stability, rather than elemental ratios alone. The combined SEM–EDX results suggest a more compact and homogeneous structure at higher filler content. The interactions between PANI and rGO are predominantly non-covalent—π–π stacking, hydrogen bonding, and mechanical interlocking—governing the enhanced structural uniformity and performance of the nanocomposites.

### Nanoindentation analysis

**Fig. 4:**
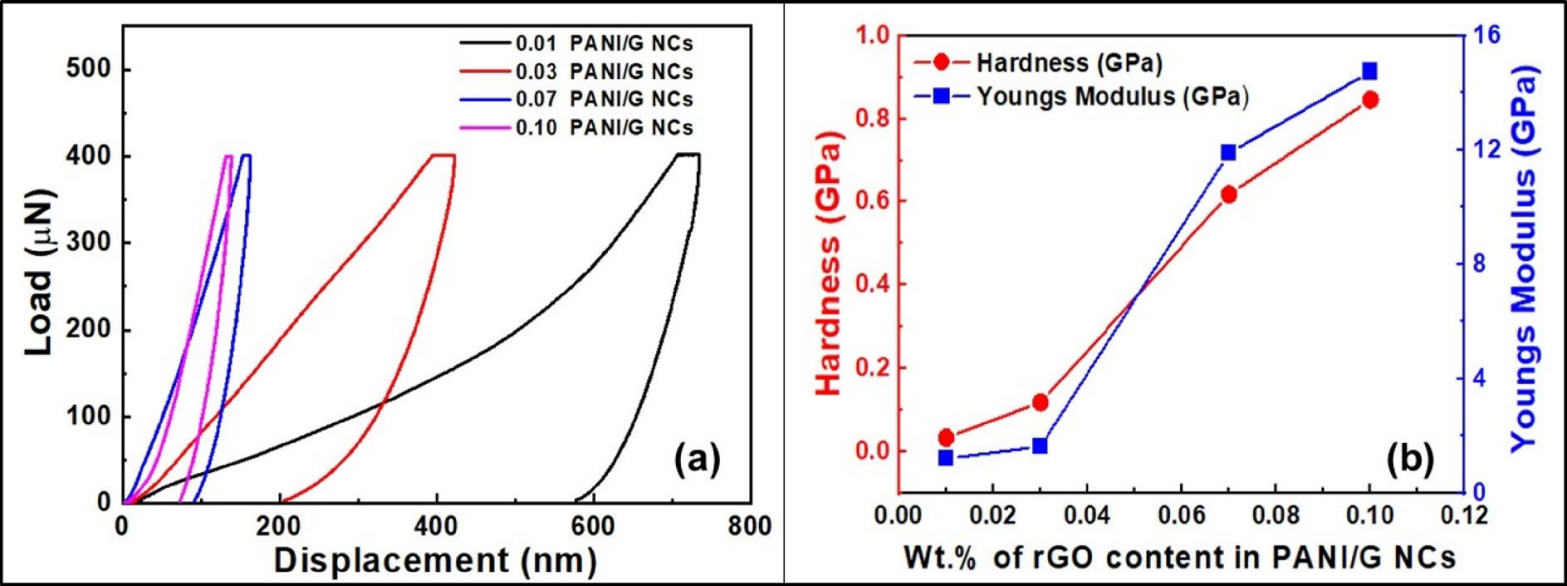
(**a**) Load v/s displacement curves obtained from nanoindentation tests, and (**b**) Variation of hardness and Young’s modulus of PANI/G NCs at rGO loadings of 0.01 wt%, 0.03 wt%, 0.07 wt%, and 0.10 wt%.

As previously discussed, nanoindentation testing was employed to evaluate the localized mechanical properties of PANI/G NCs. This method enables precise measurement of hardness and elastic modulus by recording load-displacement behavior under controlled indentation force. In the current study, a Berkovich diamond tip was used to perform nanoindentation on PANI/G NCs at a peak load of 1000 µN. Figure 4 depicts the load v/s displacement curves obtained from nanoindentation tests for PANI/G NCs at rGO loadings of 0.01 wt%, 0.03 wt%, 0.07 wt%, and 0.10 wt%. As may be observed from Fig. 4a, the curves demonstrated significant variation across the formulations. The sample containing 0.01 wt% of graphene in PANI exhibited the greatest indentation depth of ~ 750 nm, indicating its poor resistance to mechanical deformation. The indentation depth subsequently increased as the content of graphene loading increased in the samples. Furthermore, the addition of 0.10 wt% of graphene in PANI significantly reduced the penetration depth to about ~ 135 nm, reflecting enhanced stiffness and surface hardness.

Figure 4b represents the variation of hardness and Young’s modulus of PANI/G NCs at rGO loadings of 0.01 wt%, 0.03 wt%, 0.07 wt%, and 0.10 wt%. Using this information, the hardness (H) and reduced modulus (Er) were calculated using the Oliver-Pharr method^35^ given in Eqs. 1 and 2 below:

1 $$\:\varvec{H}=\frac{{\varvec{P}}_{\varvec{m}\varvec{a}\varvec{x}}}{{\varvec{A}}_{\varvec{C}}}$$

2 $$\:{\varvec{E}}_{\varvec{r}}=\frac{1}{2\surd\:\varvec{\pi\:}}\varvec{*}\frac{\varvec{S}}{\surd\:{\varvec{A}}_{\varvec{C}}}$$

Where P_max_ is the peak load, A_c_ is the projected contact area, and S is the stiffness determined from the unloading curve.

The computed values showed that the hardness for PANI/G-0.01 NCs was ~ 0.03 GPa with a modulus value of ~ 1.6 GPa, while for PANI/G-0.10 NCs, the hardness was calculated to be ~ 0.88 GPa with a modulus value of ~ 15 GPa. The enhancement in mechanical properties can be attributed to the high aspect ratio of graphene sheets, which serve as efficient load-transfer agents and hinder plastic deformation. Notably, the observed improvements in hardness and modulus arise not only from rGO reinforcement but also from reduced porosity, improved interfacial adhesion, and suppression of microstructural defects with increasing rGO loading. Similar synergistic effects have been reported in other polymer/graphene nanocomposites, where nanosheet reinforcement promotes densification and facilitates effective stress transfer across the filler–matrix interface, thereby enhancing elastic–plastic performance^25,36^. Consequently, the incorporation of graphene markedly strengthens and stiffens the PANI matrix, with the optimized PANI/G–0.10 NCs formulation exhibiting superior mechanical resistance, which is an essential attribute for high-performance applications subjected to mechanical or surface loading.

### Nanoscratch test analysis

**Fig. 5:**
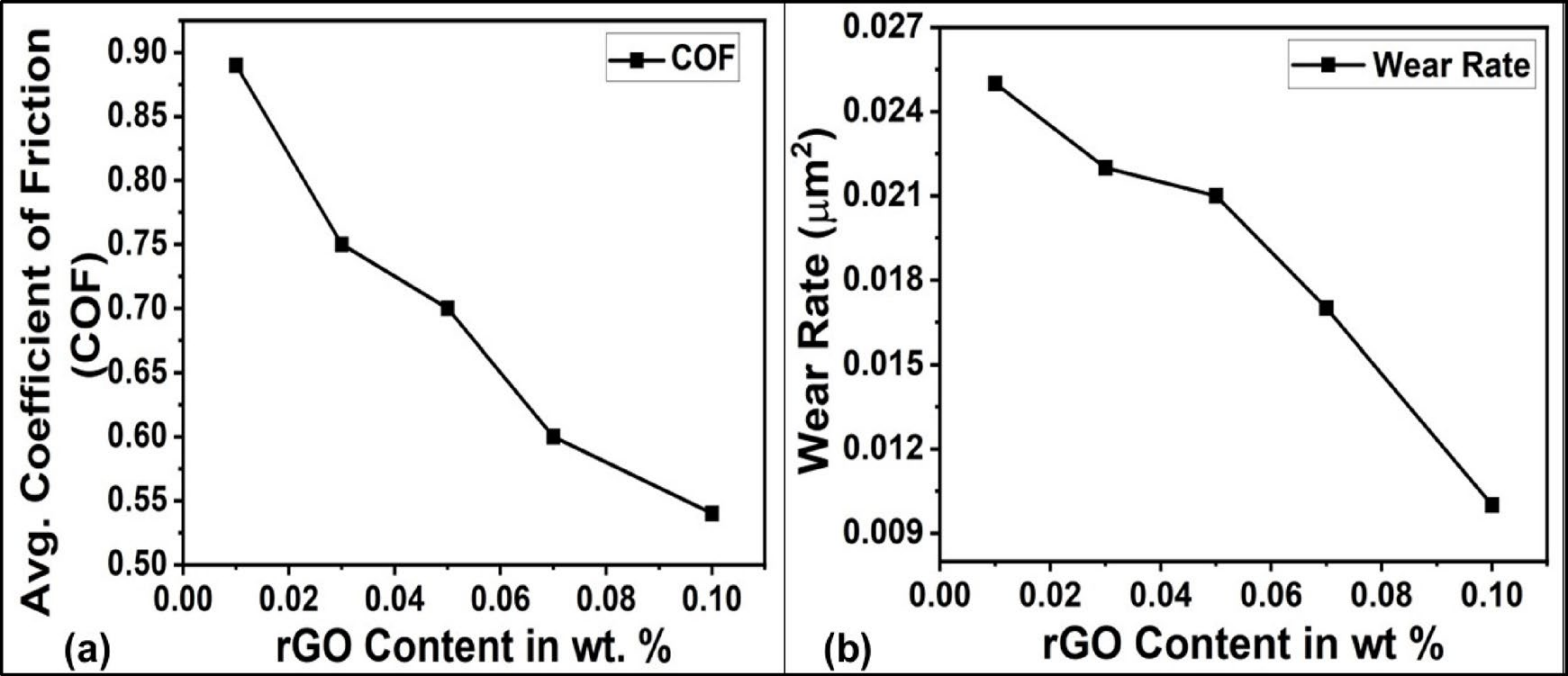
(**a**) Variation of average COF of PANI/G NCs with increasing rGO content, and (**b**) Wear rate of PANI/G NCs as a function of rGO content.

To further evaluate the surface tribological characteristics of PANI/G NCs, nanoscratch testing was performed under a constant load of 4 mN across a scratch length of 0.5 mm. The objective of carrying out the scratch analysis was to assess key tribological indicators such as the coefficient of friction (COF) and the wear rate, both of which are critical parameters in understanding the surface durability and frictional response of the as-synthesized NCs^37^.

Figure 5a represents the average COF for different rGO loadings. As may be observed from the figure, the COF decreased significantly with increasing rGO content, approximately from 0.88 for PANI/G-0.01 NCs to 0.53 for PANI/G-0.1 NCs. This trend implies a consistent reduction in interfacial friction with the increment in rGO content. Furthermore, the decrease can be ascribed to the solid lubricating nature of rGO sheets. Graphene’s two-dimensional planar structure allows for low shear resistance and easy interlayer slip, which helps in reducing the frictional forces experienced during scratching. Additionally, the improved surface uniformity and reduction of micro-asperities due to better matrix filler interaction further contribute to this decrease in COF^38^.

Figure 5b depicts the wear rate as a function of rGO content. Similar to the COF trend, the wear rate also decreased with increasing rGO loading. The highest wear rate was observed in PANI/G-0.01 NCs, containing the lowest rGO loading. On the other hand, the lowest value was recorded for PANI/G-0.10 NCs with the highest content of rGO. This reduction indicates that the incorporation of rGO strengthens the PANI matrix, facilitates more uniform stress distribution under scratch loading, and minimizes material removal. Partial surface recovery observed during progressive loading further indicates a favorable elastic–plastic balance, consistent with the H/E and H³/E² indices.

The superior tribological performance of the PANI/G-0.10 sample can be ascribed to several synergistic effects, such as matrix reinforcement, stress transfer, and solid lubrication. While PANI/G-0.10 NCs exhibited the lowest COF and wear rate, the discussion is limited to mechanistic insights rather than specific application claims, as detailed application testing (e.g., adhesion, thermal cycling) was not performed.

### Thermo-mechanical behavior

In addition to the direct hardness and modulus measurements, the elastic-plastic indices derived from nanoindentation, namely, H/E and H^3^/E^2^, provide relevant insights into the thermo-mechanical stability of PANI/G NCs. These ratios, although mechanical in origin, have direct implications for how the as-synthesized NCs withstand stress induced through thermal treatment. Typically, the H/E ratio is often correlated with elastic strain to failure and can be interpreted as a measure of the ability of the composite to recover elastically under fluctuating thermal strains. A higher H/E value suggests better accommodation for expansion and contraction without permanent damage. Likewise, the H^3^/E^2^ ratio, which reflects resistance to plastic deformation, indicates the composite’s capacity to resist irreversible deformation that may arise when repeated heating and cooling cycles introduce local stress concentrations.

**Fig. 6:**
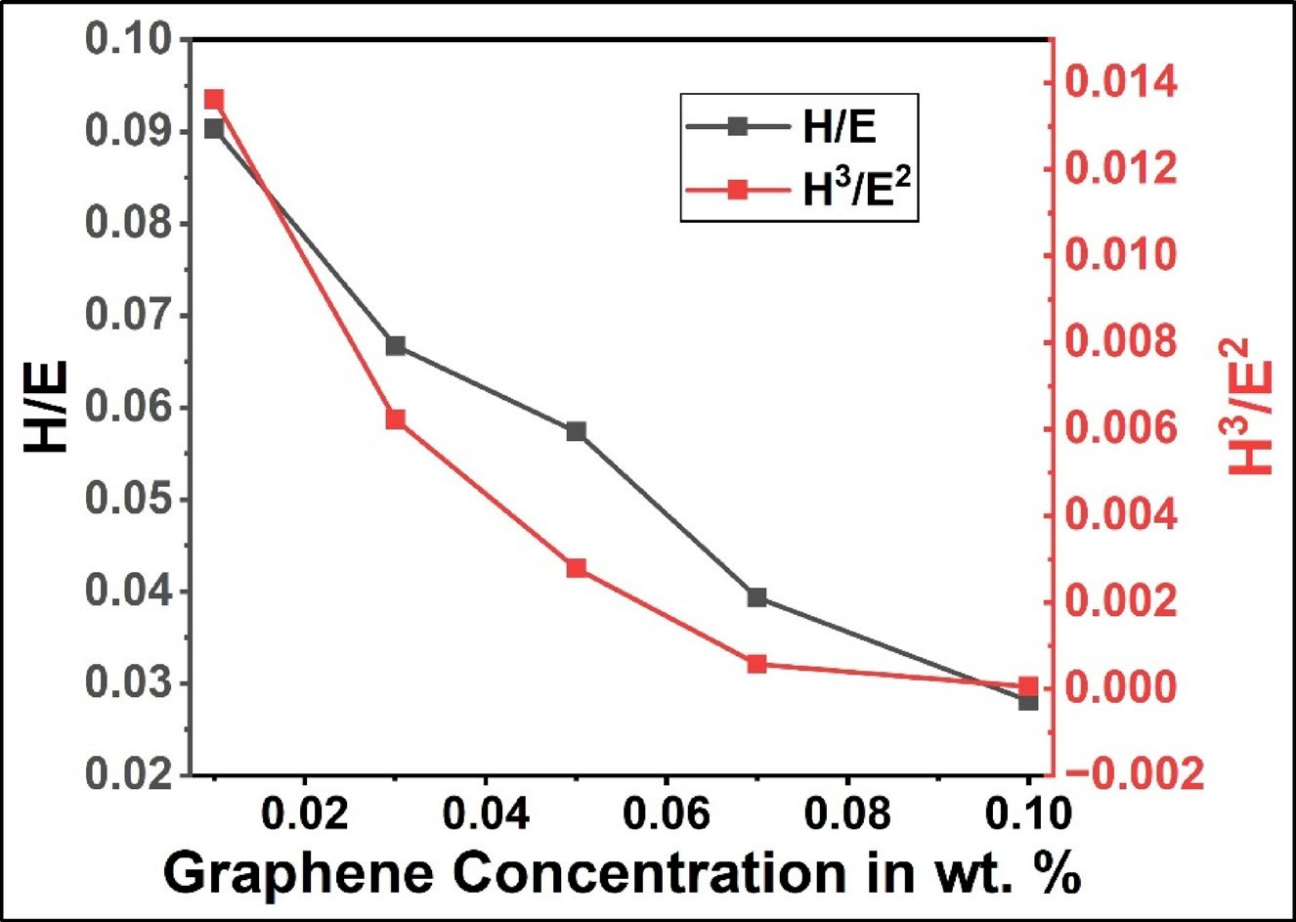
Variation of thermo-mechanical indices (H/E and H^3^/E^2^) with graphene content for PANI/G NCs.

Figure 6 represents the variation of thermo-mechanical indices (H/E and H^3^/E^2^) with graphene content for PANI/G NCs. From the figure, the observed decrease in both indices with increasing content of rGO suggests that, although stiffness and hardness increase (as shown in Fig. 4), the NCs become less capable of elastic recovery relative to their modulus. This transition implies a shift from ductile–elastic to stiffness-dominated behavior, which enhances wear resistance and dimensional stability, attributes desirable in coatings and tribological components exposed to constant surface loading or sliding contact. Conversely, moderate rGO contents (0.03–0.05 wt%) preserve a more favorable balance between stiffness and recoverability, better accommodating strain mismatches during thermal cycling or dynamic operation.

When these results are correlated with the nanoscratch and wear data, this analysis indicates that rGO’s solid-lubricating function and efficient load transfer compensate for the reduced elastic indices, yielding superior overall wear and thermal stability at 0.10 wt% rGO. Thus, *H/E* and *H³/E²* values not only describe local elastic–plastic behavior but also help rationalize the trade-offs between rigidity and flexibility crucial for tailoring PANI/rGO nanocomposites toward specific applications such as wear-resistant coatings, flexible electronics, or thermally stable sensors.

### Thermal stability from TGA

**Fig. 7:**
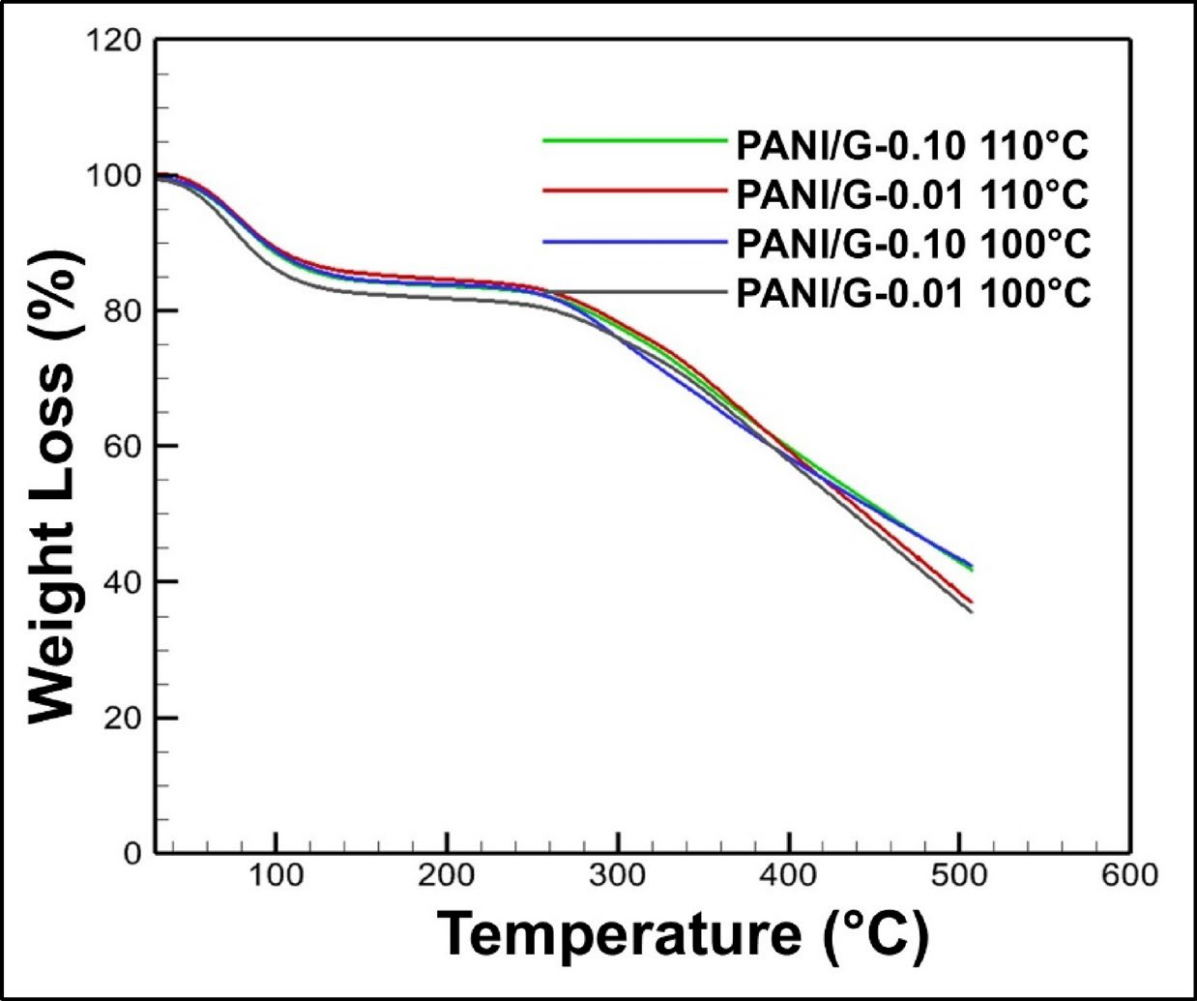
TGA graph exhibiting weight loss curves as a function of temperature for PANI/G NCs.

To further evaluate the thermo-mechanical stability, TGA was carried out for all the as-prepared samples at two processing temperatures of 100 °C and 110 °C. The results of TGA exhibiting weight loss curves as a function of temperature have been presented in Fig. 7. From the figure, it may be observed that all samples exhibit an initial weight loss below 100 °C, ascribed to the evaporation of absorbed moisture and residual solvents. A second stage of weight loss may also be observed between 150 and 250 °C, corresponding to the degradation of dopant acids and low molecular weight fragments of PANI. However, the major weight loss occurred between 300 and 500 °C, representing the backbone degradation of the PANI matrix. Notably, the PANI/G-0.10 NCs consistently displayed a delayed onset of degradation and a lower overall weight loss compared to the other samples with lower rGO loadings. This trend is evident for both annealing conditions of 100 °C and 110 °C, signifying the stabilizing role of rGO sheets in retarding thermal decomposition.

The enhancement in thermal stability may be attributed to multiple factors, such as the barrier effect of rGO sheets, which hinders the diffusion of volatile degradation products, improved interfacial interactions between PANI chains and rGO that prevent chain mobility and delay scission, and the high intrinsic thermal conductivity of rGO that facilitates heat dissipation. Remarkably, while the processing temperature (100 °C vs. 110 °C) introduced minor variations, the influence of filler concentration was far more dominant in dictating the thermal behavior.

These findings complement the previously discussed elastic-plastic indices. While those indices indicated a trade-off between stiffness and recoverability at higher rGO loading, the TGA results established that the same higher loadings (0.10 wt% rGO) imparted superior resistance against thermal degradation. Collectively, these observations highlight the multifunctional role of rGO as both a mechanical and thermal stabilizer, making the optimized PANI/G-0.10 NCs especially suitable for applications where materials are exposed to combined mechanical stress and elevated temperatures. While the TGA curves here clearly show enhanced thermal stability with increasing rGO content, the analysis remains descriptive. Detailed kinetic modeling (e.g. via Kissinger or Ozawa-Flynn-Wall approaches) was not undertaken in this study and is reserved for future work, in line with preliminary approaches in recent PANI/rGO composite studies^39^.

## Conclusions

PANI/rGO nanocomposites (0.01–0.10 wt%) were systematically studied to evaluate the effect of rGO loading on mechanical, tribological, and thermal properties. Incorporation of rGO enhanced hardness, elastic modulus, wear resistance, and thermal stability due to synergistic reinforcement, and improved interfacial adhesion.

Higher rGO loadings increased stiffness and wear resistance but reduced elastic recoverability, indicating potential brittleness, whereas moderate loadings offered a better balance between stiffness and compliance under dynamic or thermal stresses.

Although the present study identifies the optimal rGO content based on experimental trends, future work could employ statistical or machine learning (ML) based optimization to capture multidimensional dependencies among structural, mechanical, and thermal parameters. Such approaches would complement experimental insights and enable predictive design of high-performance PANI/rGO nanocomposites.

Overall, this work provides a framework linking nanoindentation indices, tribological behavior, and thermal stability, guiding rational design and further optimization of PANI/rGO systems for mechanically and thermally demanding applications.

## Data Availability

Data presented in this study are available on request from the corresponding author.
